# Transcatheter Management of Acute Mechanical Complications Post-Myocardial Infarction

**DOI:** 10.31083/RCM44308

**Published:** 2025-10-22

**Authors:** Marios Sagris, Stergios Soulaidopoulos, Konstantinos Platanias, Svetlana Aghayan, Angelos Papanikolaou, Nikolaos Ktenopoulos, Paschalis Karakasis, Konstantinos Pamporis, Athanasios Makris, Efstratios Karagiannidis, Konstantinos Aznaouridis, Nikolaos Patsourakos, Konstantinos Tsioufis, Dimitris Tousoulis

**Affiliations:** ^1^Cardiology Department, Hippokration General Hospital, School of Medicine, National and Kapodistrian University of Athens, 11528 Athens, Greece; ^2^Department of Cardiology, “Tzaneio” General Hospital of Piraeus, 18542 Piraeus, Greece; ^3^Second Department of Cardiology, Aristotle University of Thessaloniki, Hippokration General Hospital, 54642 Thessaloniki, Greece; ^4^Department of Hygiene, Social-Preventive Medicine & Medical Statistics, Medical School, Aristotle University of Thessaloniki, 54636 Thessaloniki, Greece; ^5^Department of Emergency Medicine, AHEPA University Hospital, 54636 Thessaloniki, Greece

**Keywords:** transcatheter, cardiogenic shock, myocardial infarction, TEER, VSD, aneurysm, complications

## Abstract

Mechanical complications following acute myocardial infarction (MI) represent some of the most challenging conditions in contemporary cardiology, often leading to rapid clinical deterioration and high mortality despite advances in reperfusion therapy. These complications span a spectrum of presentations, from early-phase structural disruptions such as ventricular septal rupture, papillary muscle rupture with acute mitral regurgitation (MR), and left ventricular free wall rupture (LVFWR), to later-stage manifestations, including true ventricular aneurysms and pseudoaneurysms. While surgical intervention has traditionally been considered the standard of care, surgical intervention is often associated with prohibitive risk in hemodynamically unstable or frail patients. In this context, transcatheter approaches have gained traction as viable, less invasive alternatives, offering the potential for hemodynamic stabilization, symptom relief, and improved short-term outcomes in selected patients. Nonetheless, data from observational studies and registry-based analyses remain limited, underscoring the need for further research. This review synthesizes the current evidence base and clinical experience related to transcatheter management of mechanical complications after MI, emphasizing patient selection, procedural strategies, device selection, and reported outcomes.

## 1. Introduction 

Despite significant progress in managing acute myocardial infarction (MI) over the past decades, including advances in pharmacological therapy, percutaneous intervention, and surgical reperfusion, mechanical complications remain a lethal yet under-recognized aspect of post-infarction care [1, 2, 3]. These structural sequelae, although relatively uncommon in the modern reperfusion era, are associated with high morbidity and mortality, particularly in patients with large infarcts or delayed medical intervention [1, 2].

**Fig. 1.**
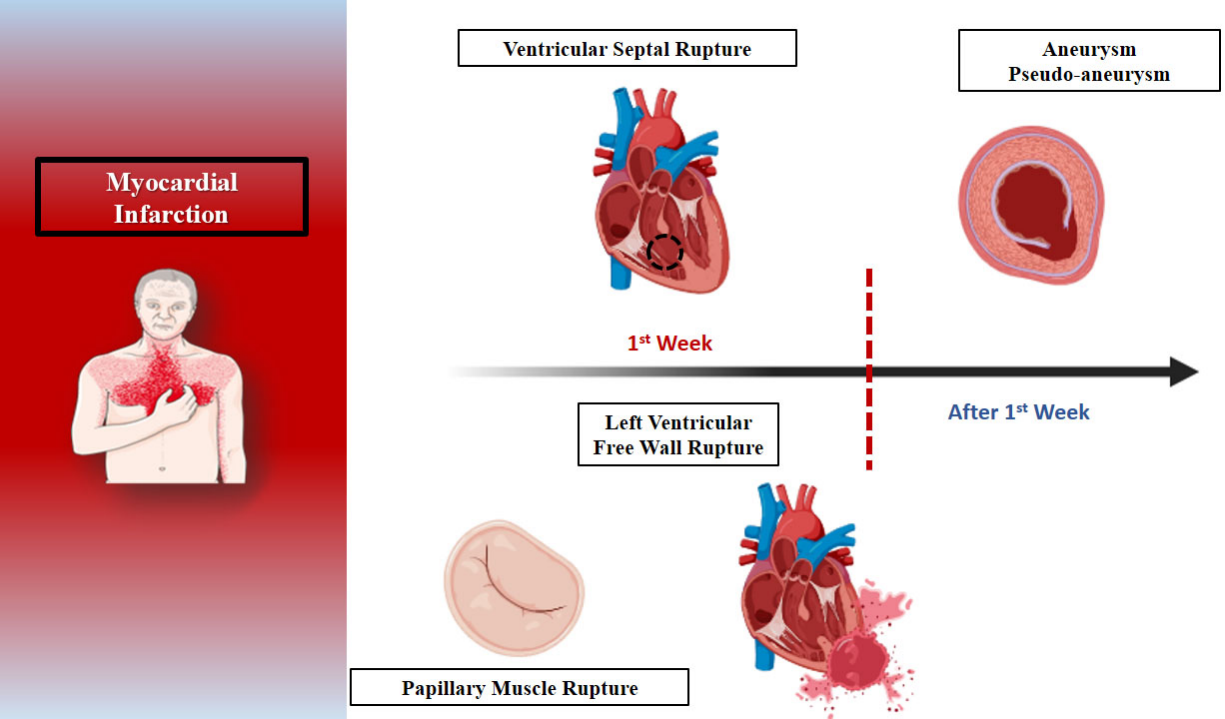
**The expected timeline of mechanical complications after myocardial infarction**.

Mechanical complications of MI span a spectrum of acute and delayed presentations, ranging from early events such as ventricular septal defect (VSD) due to rupture, papillary muscle rupture with acute mitral regurgitation (MR), and left ventricular free wall rupture (LVFWR), to late-stage consequences, such as pseudoaneurysm or true aneurysm formation [4, 5]. These complications arise from extensive myocardial necrosis and result in abrupt hemodynamic deterioration, often culminating in cardiogenic shock (CS). In the pre-reperfusion era, mechanical complications of MI such as VSD, papillary muscle rupture, and LVFWR were significantly more common, with reported incidences of 1%–2%, ~1%, and up to 4%, respectively [6, 7]. These events typically occurred within the first week following a large transmural infarct and were associated with extremely high mortality rates in the absence of prompt surgical intervention. Additionally, true left ventricular (LV) aneurysms developed in up to 35% of patients, particularly following anterior MI. However, the widespread adoption of reperfusion therapies—especially primary percutaneous coronary intervention (PCI)—has led to a significant decline in the incidence of these complications. Subsequently, VSD occurs in approximately 0.2%–0.3% of MI cases, papillary muscle rupture in approximately 0.1%–0.2%, and LVFWR in approximately 0.14%–0.3% [8, 9]. Pseudoaneurysms and true aneurysms have also become increasingly rare. Despite these advances, mechanical complications have devastating consequences, particularly in late presenters, older patients, and those without established coronary collaterals. The management remains challenging, and timely recognition is crucial to improving outcomes (Fig. 1) [8, 9].

Recently, transcatheter approaches have emerged as promising therapeutic options for select mechanical complications, providing less invasive alternatives to surgery, especially in high-risk or unstable patients. This review aims to summarize the current evidence and evolving role of transcatheter management strategies in the treatment of mechanical complications following MI, highlighting indications, techniques, outcomes, and future directions.

## 2. Papillary Muscle Rupture

Papillary muscle rupture (PMR) is a mechanical complication post-acute MI. Moreover, PMR leads to severe MR, which may be followed by pulmonary edema and CS. All those situations require emergent treatment [10]. The posteromedial papillary muscle is most commonly affected owing to its single blood supply via the posterior descending artery. Conversely, the anterolateral papillary muscle receives a dual supply from the left anterior descending and diagonal or the circumflex arteries [11]. CS represents a devastating state of diminished cardiac output and impaired end-organ perfusion, associated with increased mortality. Even in the era of early revascularization, the presence of MR in hemodynamically unstable patients with acute MI is linked to one-year mortality rates that exceed 50% [11]. MR is a potentially treatable condition and, thus, constitutes an emerging therapeutic target in patients with impaired LV contractility. The current gold standard treatment of PMR is surgery. Hence, considering that the surgical risk in patients with CS remains prohibitively high [10, 12], transcatheter edge-to-edge repair (TEER) represents a reasonable therapeutic approach. However, there remains a paucity of conclusive results about the efficacy of this method on clinical outcomes, owing to the absence of adequately sized cohorts or well-designed randomized trials.

TEER aims to approximate the anterior and posterior leaflets of the mitral valve at the origin of the regurgitant jet [11]. The deployment of TEER in the acute phase aims to reduce MR, leading to a decrease in left atrial pressure, pulmonary artery pressure, and LV volume overload, as well as an increase in cardiac output, resulting in an improvement in hemodynamic status.

In 2024, Haberman *et al*. [13] evaluated patients with post-MI PMR treated with TEER as a salvage procedure. A total of 23 patients were included, nine of them with complete papillary muscle rupture, nine with partial PMR, and five with chordal rupture. The TEER procedure was performed on a median of day 6 post-MI. The results showed that MR was significantly decreased in more than half of the patients, procedural success was achieved in 87% of the patients, while the V-Wave was reduced, and 16 out of 23 (70%) were discharged from the hospital. Five patients required reintervention with surgical mitral valve replacement. This multicenter study showed that TEER is a feasible treatment and may have a role as salvage treatment or bridge to surgery in patients with PMR and severe MR post-MI. Finally, our recent meta-analysis [14] highlights the feasibility and effectiveness of TEER in the acute phase, especially in patients with CS and severe concomitant MR. In most cases, critically ill patients who received TEER experienced considerable hemodynamic stabilization and a gradual improvement in MR to MR 2+. Meanwhile, low mortality rates, as well as MI, stroke, and re-hospitalization for HF, were recorded in both the short-term (in-hospital/30 days) and long-term (~1-year). The results were similar among the subgroup of patients presenting with CS due to acute MI, with a mortality rate of around 12% during in-hospital, which remained consistent throughout the 30-day follow-up. It is also noteworthy that the death rate remains constant throughout the follow-up period in the MI group, following the observed rise in the first 30 days, which highlights that survival during the first 30 days after the intervention is a strong predictor of long-term outcomes [14].

## 3. Ventricular Septal Defect

VSD is a serious complication that typically arises after the first 24 hours following MI. Recent studies have indicated a bimodal pattern of onset, with incidence peaks occurring on day 1 or between days 3 and 5 post-MI, underscoring the need for heightened vigilance during the initial week [4, 15]. The condition involves a rupture in the muscular portion of the interventricular septum, resulting in an abnormal communication between the left and right ventricles and frequently causing significant hemodynamic instability. Risk factors include female sex, advanced age, a first-time MI, anterior infarction, and hypertension. However, the most critical determinant remains the urgency and effectiveness of reperfusion therapy. Historically, the incidence of post-MI VSD ranged from 1% to 3% in the pre-reperfusion era; however, this has declined to between 0.17% and 0.44% following the advent of modern reperfusion techniques [5, 16].

VSD may lead to acute hemodynamic collapse primarily due to the development of a left-to-right shunt, which is characterized by increased pulmonary artery pressures and blood flow, right ventricular (RV) failure, elevated right atrial and central venous pressures, a reduction in cardiac output, and elevated pulmonary capillary wedge pressures [17]. Of course, there are several factors contributing to hemodynamic instability and its acceleration, such as defect size, RV and LV pressures and function, and pulmonary as well as systemic vascular resistance. The LV–RV function plays a crucial role; dysfunction of the LV leads to further decreased cardiac output and impaired forward flow. LV afterload is a key target for optimization; factors that increase LV afterload or decrease RV afterload will increase the left-to-right shunt, while factors that decrease LV afterload or increase RV afterload will reduce or even reverse the left-to-right shunt [18].

Management of patients in CS due to MI–VSD is a challenging case that requires the formation of a dedicated shock team, ideally including a cardiovascular intensive care physician, an interventional cardiologist with experience in structural heart disease, a cardiovascular imaging specialist, a heart failure cardiologist, and a cardiac surgeon with expertise in structural heart disease [5, 19]. The shock team should assess signs of hemodynamic compromise and propose therapeutic options to control it, applying appropriate pharmacological treatment as needed, along with the type and need for mechanical circulatory support (MCS) (intra-aortic balloon pump (IABP) is a first-line choice) [5, 19]. The in-hospital/30-day mortality rates for conservative, surgical, and interventional treatments are estimated at 94%, 44%, and 55%, respectively. Surgical VSD closure is still considered by many as the first-line therapy. However, factors such as very high surgical risk, previous unsuccessful surgical attempt, single/simple defect, a defect size <24 mm, sufficient rim margins, and adequate distance from valve apparatuses favor the transcatheter closure of VSD [20].

Successful transcatheter closure of post-infarction VSD hinges on meticulous patient selection, thorough procedural planning, and the availability of experienced operators. Subsequently, a transfer to specialized high-volume centers should be considered for stable patients. Importantly, urgent surgical intervention should be reconsidered when access to a qualified interventionalist is significantly delayed. The procedure is typically performed under transesophageal echocardiographic (TEE) guidance. Accessing the VSD is most often achieved from the arterial side, with subsequent snaring of the wire in the pulmonary artery via a venous approach. Femoral venous access is used for anterior defects, while internal jugular access is preferred for basal defects, to establish an arteriovenous rail. Device sizing is based on echocardiographic measurements, typically with a minimum of 3 mm oversizing to ensure adequate sealing of the defect. The occluder is then advanced from the venous side and delivered using a 9 or 10 French long sheath. To facilitate re-crossing of the defect in case of suboptimal deployment, a 0.018-inch wire can be left in the left ventricle [21, 22]. In selected cases, a single arterial access approach—avoiding the creation of an arterio-venous circuit—has also been reported [23].

Pre-procedural imaging using computed tomography or magnetic resonance imaging is critical for defining the anatomy, size, and dynamic nature of the defect, as VSD dimensions may vary between systole and diastole. Amplatzer occluder devices (Abbott Vascular, Santa Clara, CA, USA) are the most commonly used for this indication, featuring larger discs and a longer waist compared to standard congenital VSD occluders [24, 25]. These devices are typically deployed via the venous rail across the defect. Once positioned, TEE and contrast fluoroscopy are used to evaluate device seating and assess residual shunt before final release. Additional devices may be required in cases of persistent residual flow. Although oversizing can enhance sealing, it must be carefully balanced against the risk of damaging adjacent structures or exacerbating the defect. Reported complications include arrhythmias, ventricular rupture, device embolization, hemolysis, bleeding, stroke, and death. Device embolization occurs in approximately 7.6% of percutaneous cases, while partial and complete patch dehiscence have been reported in 13.4% and 4.3% of surgical closures, respectively [24, 25].

It is challenging to clearly illustrate the outcomes of the transcatheter approach due to the rarity of mechanical complications after MI and the inability to design adequate randomized controlled studies. The largest UK national registry included 130 patients, half of whom presented with CS, while transcatheter closure of VSD was performed in 85%. A partial and complete shunt reduction was observed in 70% and 20%, respectively, with a difference noted in 10%. In-hospital mortality was high (~55%), while complications such as device embolization (8%) and re-do procedure (13%, transcatheter or surgery) were not rare [24]. Finally, a hybrid approach (surgical/transcatheter) has been suggested for apical MI–VSD to increase the success of the procedure [26].

## 4. Pseudoaneurysms

LV pseudoaneurysm constitutes a very rare but very serious complication of MI, associated with an increased risk of mortality. The term refers to a rupture of the LV wall that is contained by pericardial adhesions or scar tissue [27]. In the majority of cases, LV pseudoaneurysm is the result of an acute MI but may also follow a cardiac surgery, a myocardial infection, or a cardiac trauma. A significant number of patients may remain asymptomatic, while symptoms related to congestive heart failure, chest pain, and dyspnea may be reported. Non-specific ST-segment changes may be observed in the electrocardiogram (ECG), while a murmur may be revealed by clinical examination in the majority of patients. However, the diagnosis is usually set with either ultrasound imaging or LV angiography in the presence of a high clinical index of suspicion [27].

Given that pseudoaneurysms are prone to rupture, the prompt management of pseudoaneurysms is crucial for preventing life-threatening complications such as cardiac tamponade, heart failure, and sudden cardiac death. The management strategy depends on the size and location of the pseudoaneurysm, as well as the clinical stability of the patient. Surgical intervention represents the gold-standard management approach, especially for large, rapidly expanding pseudoaneurysms [28]. Surgical intervention typically involves excision of the pseudoaneurysm and either direct closure of the defect or patch repair using pericardial or synthetic material. Conversely, catheter-based, minimally invasive techniques are emerging as a feasible alternative for high-surgical risk candidates [28].

The procedure is usually performed in the acute setting. Ideally, a right heart catheterization should be performed before closure to obtain a baseline assessment of the pressures in the cardiac chambers and in the pulmonary vasculature. Imaging with either transesophageal echo or more advanced techniques, namely computed tomography (CT) angiogram or cardiac magnetic resonance imaging (MRI), to assess the structure of the LV and determine the precise anatomical characteristics and the dynamic changes of the pseudoaneurysm, is of crucial importance for the planning of the procedure [29, 30]. The size and type of device to be used for closure, as well as the approach, can also be selected based on information obtained from pre-procedural imaging [31].

The concept of the procedure is to block the communication between the left ventricle and the pseudoaneurysm by implanting a closure device at the orifice of the pseudoaneurysm. A transseptal approach to the left ventricle is commonly chosen, though cases utilizing a retrograde approach through the aortic valve or a transapical approach have also been reported [30]. The transseptal approach begins with a transseptal puncture guided by transesophageal imaging. Once access to the left atrium is obtained, the pseudoaneurysm can be engaged using a 7 Fr AL-2 guide catheter, with a 0.035-inch guidewire advanced into it. After confirming access to the pseudoaneurysm with the guidewire, the AL-2 catheter is exchanged for a 5 Fr hypo tube catheter, which is positioned at the orifice of the pseudoaneurysm over the wire. The guidewire is then removed, and a vascular plug or duct occluder is advanced and deployed into the orifice of the aneurysm. The procedure is considered successful when no communication between the LV cavity and the pseudoaneurysm can be detected after the plug is deployed. The patient remains hospitalized for 1–2 days after the procedure, unless there is another reason to prolong hospitalization. Accordingly, in the retrograde approach, access to the LV cavity is achieved through the aorta [30].

Potential periprocedural complications include bleeding, hematoma, infection, embolism of the closure device, arrhythmia, pacemaker dependence, stroke, and/or death. Alternatively, the presence of a left atrial thrombus, active endocarditis, and cardiac anatomy unfavorable to catheter intervention should be considered contraindications for performing the procedure [31].

## 5. Aneurysms

Left ventricular aneurysms (LVAs) represent a rather infrequent complication of transmural MI. In contrast to LV pseudoaneurysms, LVA are broad-necked, discrete, dyskinetic areas in the LV wall resulting from the fibrotic replacement of necrotic myocardium following MI. These distort cardiac geometry and function and are associated with the development of various devastating complications, including congestive heart failure, thrombus formation, thromboembolism, and malignant ventricular arrhythmias [32, 33].

The choice of treatment is based on the size of the LVA and on the extent to which it affects cardiac function. On the one hand, the conservative approach includes the administration of drugs such as β-blockers, angiotensin-converting enzyme inhibitors, and statins, aiming to limit ventricular dilatation, improve clinical symptoms, prolong survival, and improve the quality of life [34]. Conversely, a more aggressive strategy involving surgical ventricular reconstruction through resection of the LVA, typically combined with coronary artery bypass graft (CABG), may be considered for selected patients. Notably, the STICH trial demonstrated that combining CABG with ventricular reconstruction offers a survival benefit over CABG alone in patients with coronary artery disease suitable for revascularization, who also have reduced LV systolic function (EF ≤35%) and exhibit anterior wall akinesia or dyskinesia [35]. Similarly, catheter-based procedures for direct modification of the left ventricle are gaining traction and offer an alternative strategy for the interventional management of large infarct aneurysms.

The Revivent TC devices (BioVentrix Inc., San Ramon, CA, USA) enable the application and exclusion of the LVA using paired micro-anchors, which are applied through a hybrid procedure. One anchor is surgically implanted into the scarred epicardial surface of the LV epicardium through a 4 cm thoracotomy; meanwhile, the other is introduced percutaneously into the right side of the interventricular septum using a catheter advanced through the venous system. Specifically, a snare catheter is advanced into the right ventricle via the internal jugular vein to capture a wire that has been passed through a needle inserted via a small thoracotomy, traversing the anterior wall of the left ventricle and the interventricular septum. Once snared, the wire is drawn through the jugular vein. An internally hinged anchor is then loaded onto the wire and advanced to the right side of the interventricular septum [36]. Notably, two to three pairs of anchors are usually implanted to achieve a sufficient area of scar exclusion and volume reduction. The plication of the scarred myocardial tissue is achieved by clinching the anchors together, and, in this way, the non-functional, scarred part of the LV is excluded [37]. Compared to the surgical method, the Revivent TC procedure requires no sternotomy, no ventriculotomy, and no extracorporeal or circulatory support. Data regarding the efficacy of the procedure are currently limited and primarily derived from single-center studies. A prospective study involving 26 patients who underwent the procedure between 2017 and 2019 demonstrated a significant increase in left ventricular ejection fraction (LVEF), along with a notable reduction in LV volume 9 months after the intervention. These changes were accompanied by a marked improvement in functional status, as assessed by the 6-minute walking test and the New York Heart Association (NYHA) classification of heart failure. In terms of hard endpoints, one patient died, and another required three re-hospitalizations due to recurrent heart failure [38].

Percutaneous restoration therapy with the use of the Parachute device (Cardiokinetix, Menlo Park, CA, USA) offers another minimally invasive alternative to surgery for the interventional treatment of patients with LV antero-apical wall motion abnormality. The Parachute device consists of a self-expanding nitinol frame (16 struts; radio-opaque), an ePTFE impermeable membrane, and an atraumatic polymer foot available in four sizes (65, 75, 85, and 95 mm) with two different “foot” heights. The device is anchored on the myocardium through the tips of the struts; meanwhile, the atraumatic foot connects the LV apex with the device and allows orientation of the device with a vector towards the outflow tract [39]. The concept of percutaneous ventricular restoration is based on the hypothesis that the placement of a partitioning, compliant device at the LV apex may achieve geometric configuration, synchronized wall motion, and volume reduction of the LV, ultimately restoring and improving the LV systolic function [40]. Previous trials evaluating the efficacy of the technique have shown a procedural success rate exceeding 90% for the device implantation. In addition to this, over a three-year follow-up period, the device has demonstrated significant benefits on LV hemodynamics, including reduced LV volumes and increased LV ejection fraction, accompanied by improved functional status in patients with ischemic heart failure [41, 42].

While not conclusive, the favorable long-term outcomes observed in this high-risk population provide encouraging evidence for the safety and feasibility of both aforementioned techniques, supporting the need for further investigation of this novel therapeutic approach.

## 6. Left Ventricular Free Wall Rupture

LVFWR is a devastating mechanical complication that occurs in approximately 2–4% of patients following acute MI, typically within the first week. The condition carries an extremely high mortality rate and is traditionally managed as a surgical emergency. Open-heart surgical repair remains the gold standard due to the need for definitive anatomic correction and control of hemorrhage [43]. However, in cases where patients are deemed unsuitable for surgery due to profound hemodynamic instability, advanced age, or comorbid conditions that predict poor perioperative outcomes, percutaneous intervention has emerged as a potential life-saving alternative or as a temporizing bridge to surgery [44].

In the absence of pseudoaneurysm formation, free wall rupture leads to rapid accumulation of blood in the pericardial space, resulting in pericardial tamponade and cardiovascular collapse. Prompt diagnosis is paramount and hinges on the use of transthoracic and transesophageal echocardiography, which helps identify pericardial effusion and suggest active rupture [43].

Indications for percutaneous management [44]:

∙ Hemodynamic instability, which represents a critical condition for surgical intervention.

∙ Sudden clinical deterioration despite pericardiocentesis.

∙ Limited rupture visualized on echocardiography.

∙ High predicted surgical risk.

The percutaneous technique involves initial pericardial drainage to stabilize the patient, followed by transcatheter closure using devices such as the Amplatzer Septal Occluder or the Amplatzer VSD Occluder [45]. These devices are typically deployed via femoral access under combined fluoroscopic and echocardiographic guidance. A guidewire is maneuvered across the rupture site, and the occluder is positioned such that one disc lies within the LV cavity and the other in the pericardial space, effectively sealing the rupture. Success is confirmed by cessation of bleeding and hemodynamic improvement, as visualized by real-time imaging [45].

Although experience with percutaneous closure of LVFWR remains limited, early case reports and small case series suggest that this approach can be technically feasible and clinically effective in select patients [44]. The intervention is best considered in the context of a multidisciplinary heart team, particularly when surgery is not immediately available or carries unacceptable risk. While the procedure is complex and demands significant operator skill, it holds promise as an emergent intervention in high-risk patients. Future developments in device technology and procedural standardization, alongside broader clinical experience, will be essential in establishing percutaneous repair as a validated strategy for managing this lethal complication [44, 45, 46].

## 7. Role of Mechanical Circulatory Support 

MCS has been introduced as a management option in addition to conservative treatment, primarily as a bridge to definitive therapy. Data from the National Inpatient Sample (2016–2020) show that MCS was used in 44.3% of mechanical complication cases, with a rising trend in usage over time (39.3% in 2016 to 48.9% in 2020), although without a corresponding improvement in mortality (36.9% in 2016 vs. 43.4% in 2020). The IABP was the most commonly used device, likely due to its greater availability, lower cost, and ease of use. In contrast, the limited use of advanced devices, such as Impella and VA-ECMO, precluded meaningful comparisons [47]. MCS use was more frequent in younger, male patients with fewer comorbidities, indicating possible selection bias toward those with a more favorable prognosis. While MCS was associated with longer hospital stays, it was linked to improved survival in the PMR and pseudoaneurysm subgroups; however, no overall survival benefit was observed. Mortality remained high in both the MCS and non-MCS groups (48.4% vs. 34.5%, respectively), underscoring the need for further investigation to define the role of MCS in managing mechanical complications after MI [48]. Currently, consensus statements propose the use of IABP as a first-line option for patient stabilization [20]. However, there is a lack of randomized controlled trials in this domain, primarily due to the rarity of the condition and the differences in hemodynamic performance among entities. As such, there is a clear need for individualized treatment and a meticulous study of the hemodynamic effects of each mechanical complication subtype to select the appropriate device support.

The involvement of a dedicated shock team is crucial in managing refractory CS. This team should ideally be multidisciplinary, bringing together a cardiovascular intensive care physician, an interventional cardiologist experienced in structural heart interventions, a cardiovascular imaging specialist, a heart failure cardiologist, and a cardiac surgeon skilled in complex structural procedures. When local expertise or resources are lacking and advanced therapies are required, the patient should be considered for protected transfer, supported by temporary MCS, to a center equipped with these capabilities. If transfer is not feasible and both surgical and percutaneous options are deemed futile, the shock team should initiate a discussion on transitioning to palliative care [19].

## 8. Conclusion 

Mechanical complications following acute MI can severely compromise hemodynamic stability and are associated with extremely high mortality. In this critical setting, for patients deemed inoperable or at prohibitive surgical risk, transcatheter interventions may offer a life-saving alternative, with emerging evidence suggesting promising outcomes.
